# Highly Stable Porous Polyimide Sponge as a Separator for Lithium-Metal Secondary Batteries

**DOI:** 10.3390/nano10101976

**Published:** 2020-10-06

**Authors:** Junyoung Choi, Kwansoo Yang, Hyeon-Su Bae, Isheunesu Phiri, Hyun Jeong Ahn, Jong Chan Won, Yong Min Lee, Yun Ho Kim, Myung-Hyun Ryou

**Affiliations:** 1 Department of Chemical and Biological Engineering, Hanbat National University, 125 Dongseo-daero, Yuseong-gu, Daejeon 34158, Korea; jychoi@krict.re.kr (J.C.); hsbae1234@gmail.com (H.-S.B.); isheunesuphiri@gmail.com (I.P.); 2 Advanced Materials Division, Korea Research Institute of Chemical Technology (KRICT), 141 Gajeongro, Yuseong-gu, Daejeon 34114, Korea; sky214@krict.re.kr (K.Y.); jj0223@krict.re.kr (H.J.A.); jcwon@krict.re.kr (J.C.W.); 3 Korea Research Institute of Chemical Technology (KRICT) School, University of Science and Technology, 217 Gajeongro, Yuseong-gu, Daejeon 34113, Korea; 4 Department of Energy Science and Engineering, Daegu Gyeongbuk Institute of Science and Technology (DGIST), 333 Techno Jungang-Daero, Daegu 42988, Korea

**Keywords:** Li-metal electrode, Li secondary batteries, polyimide separators, water-soluble poly(amic acid), porous polymer

## Abstract

To inhibit Li-dendrite growth on lithium (Li)-metal electrodes, which causes capacity deterioration and safety issues in Li-ion batteries, we prepared a porous polyimide (PI) sponge using a solution-processable high internal-phase emulsion technique with a water-soluble PI precursor solution; the process is not only simple but also environmentally friendly. The prepared PI sponge was processed into porous PI separators and used for Li-metal electrodes. The physical properties (e.g., thermal stability, liquid electrolyte uptake, and ionic conductivity) of the porous PI separators and their effect on the Li-metal anodes (e.g., self-discharge and open-circuit voltage properties after storage, cycle performance, rate capability, and morphological changes) were investigated. Owing to the thermally stable properties of the PI polymer, the porous PI separators demonstrated no dimensional changes up to 180 °C. In comparison with commercialized polyethylene (PE) separators, the porous PI separators exhibited improved wetting ability for liquid electrolytes; thus, the latter improved not only the physical properties (e.g., improved the electrolyte uptake and ionic conductivity) but also the electrochemical properties of Li-metal electrodes (e.g., maintained stable self-discharge capacity and open-circuit voltage features after storage and improved the cycle performance and rate capability) in comparison with PE separators.

## 1. Introduction

Portable electronic devices such as cellphones and laptops have revolutionized modern society. Li-ion batteries (LIBs), which power mobile electronic devices, have been crucial for this revolution [1,2,3]. Three scientists who each contributed to pivotal developments in Li-ion battery technology received the 2019 Nobel Prize in Chemistry, which further supports this contention.

To make the Earth a clean and sustainable planet, a prerequisite is to reduce fossil-fuel-based power generation. To that end, LIBs are on the verge of transforming the transportation and energy-storage sectors by enabling the development of efficient electric vehicles (EVs) and energy storage systems (ESSs). State-of-the-art LIBs are based on Li-transition metal oxides and graphites. Large-scale electrical applications such as EVs and ESSs require high-energy-density batteries, whose performances surpass those of traditional LIBs. Thus, the development of new electrode systems has attracted immense attention. Because the energy density of batteries is determined by the product of capacity and operating voltage (i.e., energy = voltage × capacity), Li metal, which has the highest theoretical capacity (3860 mAh g^−1^) and the lowest operating potential (−3.04 V versus standard hydrogen electrode) among the candidates for anode active material, has been considered as a promising anode active material [4].

Owing to the severe capacity deterioration and safety issues of Li metal ascribed to Li-dendrite formation, Li-metal electrodes have not been successfully implemented for the development of commercial Li secondary batteries. Li-dendrite formation has been regarded as an inherent property of Li-metal electrodes, and numerous researchers have attempted to overcome the critical drawbacks of Li-metal electrodes using the following methods: (1) morphological modification of the Li metal [5,6,7], (2) electrolyte modification using functional additives [8,9,10,11], (3) protective layers [12,13,14], and (4) separator modification [4,15,16].

Microporous separators are considered as a physical barrier between the anode and cathode. However, the role of separators in inhibiting Li-dendrite growth has been underestimated. Since we first suggested that a uniform current distribution controlled by separators can effectively reduce Li-dendrite formation [4,17], several studies have reported the modification of microporous separators. In terms of uniform current distribution, three-dimensionally ordered macro-porous (3DOM) separators are promising candidates that can control current distribution through precise pore control [18,19,20,21].

Recently, porous polyimide (PI) separators with 3DOM structures that provide uniformly deposited Li have been reported [20,21,22]. PI is a typical thermal resistance engineering polymer with exceptional thermal and chemical stability and excellent mechanical properties. When compared with other conventional thermoplastic polyolefin-based separators, which generally have a melting temperature below 160 °C, thermally resistant PI separators are advantageous in preventing short-circuits and explosions at high temperatures by suppressing dimensional changes [23,24,25,26,27,28]. Nevertheless, 3DOM-PI separators have several disadvantages: (1) Their manufacture has low productivity and high production cost. In general, 3DOM-PI separators are prepared by the colloidal crystal templating method using silica (SiO_2_) particles, which incurs a high processing cost owing to the complicated process. Once the closely packed SiO_2_ particle superlattices embedded in the polymer matrix are formed, SiO_2_ particles are selectively removed using hydrofluoric acid to build inverse-opal polymer separators. (2) Difficulty in controlling separator thickness. The thickness of the 3DOM-PI separators is mainly determined by the number of SiO_2_ particle layers, which is complicated to manage. (3) Environmentally unfriendly preparation process. In general, PI separators are prepared by coating polyamic acid solution dissolved in organic solvents such as *N*-Methyl-2-pyrrolidone (NMP), dimethylformamide, and *N*,*N*-dimethylacetamide, followed by a thermal imidization process [23,25,26,27,28].

In this study, we propose a simple and environment-friendly method for preparing high-temperature stable PI sponge separators for Li-metal electrodes using a solution-processable high internal-phase emulsion (HIPE) technique with a water-soluble PI precursor solution. Through a simple solution process, HIPEs can be used as templates to produce highly porous polymeric materials [29]. We adopted the environmentally friendly water-soluble PI precursor solution as a continuous aqueous phase to prepare a stable oil-in-water (O/W) HIPE. PI sponges with an open-cell porous structure were obtained from the PI-precursor-based HIPE through a process that included freeze-drying, thermal imidization, and subsequent pressing. The physical properties (e.g., thermal stability, wettability to liquid electrolytes, and ionic conductivity) of the free-standing PI sponge-based separators (hereafter, referred to as porous PI separators for convenience) and their effect on the electrochemical properties (e.g., cycle performance and rate capability) of Li-metal anodes were investigated. In addition, the performance of commercialized PE separators was compared with that of porous PI separators.

## 2. Materials and Methods

### 2.1. Materials

Pyromellitic dianhydride (PMDA, purity = 97%) and 2,2′-dimethylbenzidine (DMBZ, purity = 98%) were purchased (Sunlight Pharmaceutical Co., Ltd., Changzhou, China) and used after drying in a vacuum oven for 24 h at 60 °C. We purchased 1-methyl-2-pyrrolidinone (NMP, purity = 99.5%), dimethylethanolamine (DMEA, purity = 99.5%), and cyclohexane (purity = 99%) from Sigma–Aldrich (Seoul, Korea) and used them as received without further purification. Li-metal foil (thickness = 200 μm, Honjo Metal, (Osaka, Japan), Li cobalt oxide (LiCoO_2_, KD-10, Umicore, (Cheonan, Korea), conductive carbon (Super-P Li; Imerys, (Bironico , Switzerland), polyvinylidene fluoride (PVdF, KF–1300; Kureha, (Tokyo, Japan; M*w* = 350,000), and *N*-methyl-2-pyrrolidone (NMP, anhydrous, purity = 99.5%, Sigma–Aldrich, Seoul, Korea) were used without additional treatment. A mixture of 1.15 M Li hexafluorophosphate (LiPF_6_) in ethylene carbonate/ethyl methyl carbonate (EC/EMC = 3/7, by vol.) was used as a liquid electrolyte without additional purification (Enchem Co., Ltd., Cheonan, Korea). Microporous polyethylene (PE) separators (thickness = 20 μm, ND420, Asahi Kasei E-materials, Tokyo, Japan) were used as reference separators.

### 2.2. Porous PI Separator Preparation

Water-soluble poly(amic acid) (PAA) based on PMDA-DMBZ was prepared following the methods explained in our previous reports [30]. Notably, to prepare an aqueous PAA solution, DMEA was used as a water dissolution agent [31]. The aqueous solution was prepared by adding DMEA with a double amount of a repeating unit of PAA. To fabricate porous PI sponge via the solution process, oil-in-water (O/W) HIPE was prepared with PAA aqueous solution in the water phase and cyclohexane in the oil phase (2/8 by vol.). First, a third of cyclohexane was added to the 3 wt.% PAA aqueous solution and emulsified at 25,000 rpm for 3 min by a homogenizer (T25B, IKA, Seoul, Korea). Then, the remaining cyclohexane was added in two portions and emulsified for 10 min to obtain HIPE. The prepared HIPE was filled in a silicone mold of 3 mm thickness, was freeze-dried for 24 h (Operon, FDUT-6002, Gimpo, Korea), and then thermally imidized at increasing temperatures of 120, 180, 250, 300, and 350 °C for 30 min in a vacuum oven. Finally, the porous PI separators with 20 μm thickness were fabricated by pressing the PI sponge at room temperature (25 °C) for 3 min under 10 MPa.

### 2.3. Physical Properties of Separators

The ionic conductivity of separators was measured using 2032-type coin cells, in which stainless steel (SUS) was used as the blocking electrode. Separators (diameter = 18 mm) were sandwiched between SUS electrodes, and 600 μL of liquid electrolyte was injected. The following equation determined ionic conductivity (σ), *σ = l/RS*, where R is the bulk resistance (R) measured by an impedance analyzer (VSP, BioLogic Co., France), *l* is the thickness of separators, and S is the contact area between separators and SUS blocking electrodes [32].

The uptake amount of separators was calculated using the following equation, uptake amount (wt.%) = (W_2_ − W_1_)/W_1_ × 100, where W_1_ and W_2_ indicate the weights of the separators (size = 3 × 3 cm) before and after absorbing the liquid electrolyte (12 h, 25 °C), respectively [33].

### 2.4. Electrode Preparation

Li-metal foil was cut using a hollow hole punch and employed as a Li-metal electrode (radius = 1.2 cm). LiCoO_2_ cathodes were fabricated by casting an NMP-based slurry comprising 90 wt.% of active material (LiCoO_2_), 5 wt.% polymeric binder (PVdF), and 5 wt.% conductive carbon (Super-P) on an Al foil (15 µm, Sam-A Aluminum, Korea) using a gap-controlled doctor blade and were then dried in a convection oven for 1 h at 130 °C. After drying, the LiCoO_2_ cathodes were calendared with a gap-control-type roll-pressing machine (CLP-2025, CIS, Daegu, Korea) and dried overnight in a vacuum for 12 h at 70 °C. The loading level, density, and thickness of LiCoO_2_ cathodes were 12 mg cm^−2^, 1.8 g cm^–3^, and 64 μm, respectively.

### 2.5. Cell Assembly

We then assembled 2032 coin-type cells in a glove box filled with argon gas with a dew point below –60 °C at 25 °C. For Li-metal half cells (LiCoO_2_/separator/Li metal), separators (diameter = 18 mm) were sandwiched between LiCoO_2_ (diameter = 12 mm) and the Li metal (diameter = 15 mm). For Li–Li symmetric cells, separators (diameter = 18 mm) were sandwiched between Li electrodes of different diameters (diameter = 12 and 15 mm). For both cases, every constituent (LiCoO_2_, separator, and Li metal) was soaked in the liquid electrolyte for 12 h at 25 °C.

### 2.6. Electrochemical Analysis

Galvanostatic cycling for Li–Li symmetric cells (Li metal/separator/Li metal) was performed based on an iterative loop: +0.5 mA cm^−2^ (30 min) → Rest (10 min) → –0.5 mA cm^−2^ (30 min) → Rest (10 min). The 10 min rest between plating and stripping processes is required to diminish the influence of concentration gradients.

The precycling procedure for Li metal half cells (LiCoO_2_/separator/Li metal) included one formation and three stabilization cycles. Every cycle was performed in a potential window between 3.0 and 4.3 V. For the formation cycle, Li metal half cells were charged and discharged in a constant current (CC) mode at C/10 (current density = 0.12 mA cm^−2^). For the stabilization cycles, Li metal half cells were charged in a CC/constant voltage (CV) mode and then discharged in a CC mode at C/5 (current density = 0.24 mA cm^−2^). Cycle performance of the Li metal half cells was evaluated in a CC/CV mode for the charging process (1C, 1.2 mA cm^−2^) and in a CC mode for the discharging process (1C, 1.2 mA cm^−2^) at 25 °C using a charge–discharge cycle tester (PNE Solution, Suwon, Korea). The rate capability of Li metal half cells was evaluated in a potential window between 3.0 and 4.3 V with varying discharge current densities (C/2 = 0.6 mA cm^−2^, 1C = 1.2 mA cm^−2^, 3C = 3.6 mA cm^−2^, 5C = 6.0 mA cm^−2^, 7C = 8.4 mA cm^−2^, and C/2) while maintaining the same charge current density at C/2 (0.6 mA cm^−2^).

### 2.7. Self-Discharge Property Evaluation

To evaluate the self-discharge properties, Li-metal half cells (LiCoO_2_/separator/Li metal) were precycled and then fully charged to 4.2 V (at a CC/mode, C/2 = 0.6 mA cm^−2^, 25 °C). The open-circuit voltage and discharge capacity of the fully charged Li metal half cells were measured every week. After measuring the discharge capacity, Li metal half cells were again fully charged to 4.2 V (at a CC/mode, C/2 = 0.6 mA cm^−2^, 25 °C).

### 2.8. Scanning Electron Microscopy (SEM) Analysis

All samples were analyzed using a field-emission scanning electron microscope combined with energy-dispersive X-ray analysis (FE-SEM/EDX; S-4800; Hitachi, Tokyo, Japan). To analyze the surface morphology of Li-metal anodes, coin cells were disassembled inside a glove box and washed thoroughly with dimethyl carbonate, followed by drying overnight in a vacuum (at 25 °C).

## 3. Results and Discussion

**Figure 1:**
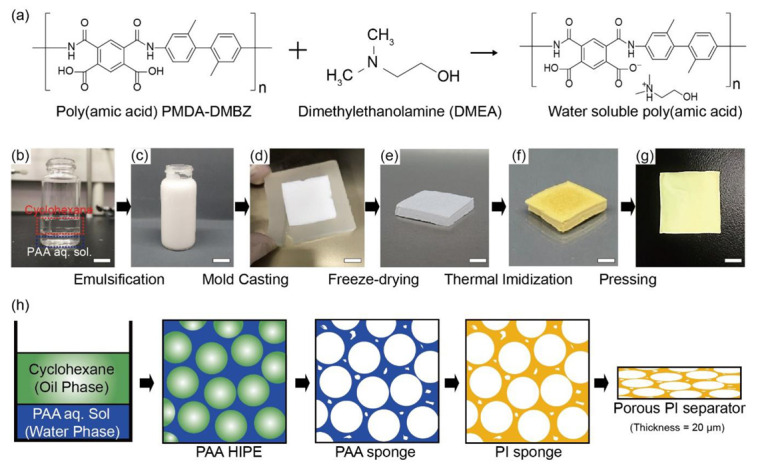
(**a**) Molecular structures of pyromellitic dianhydride - 2,2′-dimethylbenzidine (PMDA-DMBZ)-based water-soluble poly(amic acid) (PAA) and dimethylethanolamine (DMEA), (**b**–**g**) the overall fabrication procedure of porous polyimide (PI) separator using oil-in-water (O/W) high internal-phase emulsion (HIPE) and subsequent pressing, (**h**) the schematic diagram of the internal structure of each step in the order of solution, PAA-HIPE, PAA-sponge, PI sponge, and the porous PI separator. All scales in (**b**–**g**) are 1 cm.

The preparation process of porous PI separators based on an oil-in-water (O/W) HIPE technique is described in Figure 1. HIPEs generally have a high volume–fraction (Ø > 74%) of dispersed droplets in a low-volume–fraction continuous phase. They are widely employed to fabricate porous polymers by curing the monomers and polymer precursors in a continuous phase with drying of the solvents of each phase [34].

In this study, PAA, as a precursor of PI, was dissolved in the continuous water phase of HIPE in the salt phase using a specific alcohol amine (dimethylethanolamine (DMEA), here). The molecular structures of PMDA-DMBZ-based PAA and DMEA are shown in Figure 1a. The incorporation of a suitable organic base, including DMEA, trimethylamine, and 1,2-dimethylimidazole, prevents the dissociation of PAA by water molecules by forming ammonium salt with the carboxyl group (–COOH) of PAA and provides excellent water solubility as well as a significant enhancement in the hydrolytic stability [35]. Cyclohexane was used as an oil phase corresponding to 80 vol.% of the emulsion. As the freezing point of cyclohexane of 6.5 °C is similar to that of water of 0 °C, cyclohexane helps in maintaining the structural stability without phase separation (or inversion) during the whole fabrication process. Stable PAA-based HIPE was produced by emulsifying unmixed cyclohexane and an aqueous PAA solution with a high-speed homogenizer (Figure 1b,c). The ionic salt phase of PAA stabilized the oil and water interface without phase inversion at a high volume–fraction of dispersed oil droplets and allowed micrometer-sized oil droplets to be well-dispersed in the continuous phase (Figure 1c). We then freeze-dried the HIPEs within a silicone mold to maintain its porous structure and thermally imidized the remaining PAA sponge to create a PI sponge (Figure 1d–f). Subsequently, we pressed the porous PI sponge at room temperature to manufacture a porous PI separator, which could be determined by controlling the thickness of the initial PI sponge (Figure 1g). Typically, a 1.6 mm thick PI sponge can be pressed at room temperature with a pressure of 10 MPa to form a porous PI separator with a thickness of 20 μm. Figure 1h shows a schematic diagram of the internal structures of each step in the order of solution: PAA-HIPE, PAA sponge, PI sponge, and PI sponge separator.

**Figure 2:**
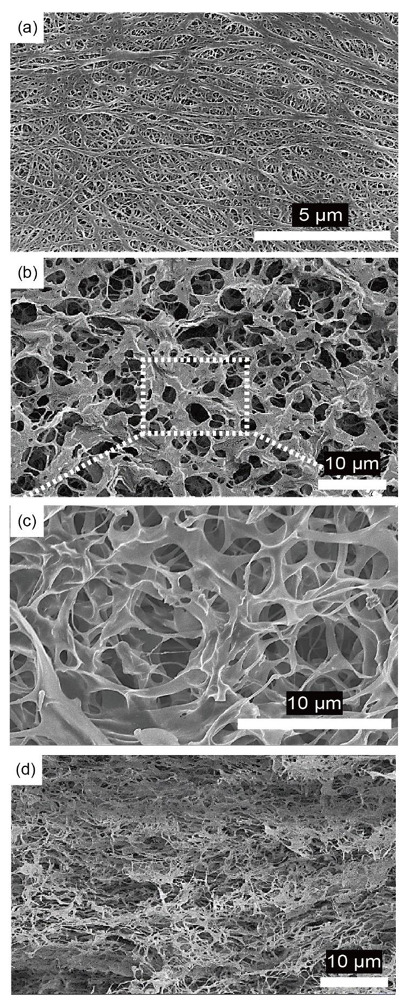
Surface scanning electron microscopy (SEM) images of (**a**) bare polyethylene (PE) separators and (**b**,**c**) porous PI separators. The cross-sectional SEM image of (**d**) porous PI separators.

First, the physical properties such as morphological structure, ionic conductivity, and liquid electrolyte uptake of the porous PI separators were investigated and compared with those of the PE separators. The surface morphology of the porous PI separators was observed using SEM. Compared with bare PE separators (Figure 2a), porous PI separators have a more porous structure (Figure 2b,c). In general, an excessively large porous structure is not suitable for materials to act as separators, especially for Li-metal anodes, owing to issues such as short-circuit and self-discharging. Li dendrites, which form from the surface of the Li-metal anodes during repeated cycling, can penetrate interconnected pores and can finally contact cathodes [36]. Therefore, the cross-sectional images of porous PI separators were observed using SEM. An open-cell type porous structure can be observed in the cross-sectional SEM image of the porous PI separator (Figure 2d). The average pore diameter of the PI sponge, measured by mercury porosimetry, was 4.6 μm, and the porosity reaches 94.9% (Supplementary Materials Figure S1 and Table S1). After pressing, the volume of the porous PI separator decreased to ca. 1/80 from PI sponge, and the pore diameter significantly reduced to less than 1 μm, as shown in Figure 2d. Furthermore, the networked open-cell porous structure was maintained as it was prepared, and these networked porous structures could effectively prevent the formation of vertically growing Li dendrites [37]. Because PI is physically and electrochemically stable, the porous PI separator was sufficiently applicable as a separator for Li-metal anodes. The effect of the porous PI separator on the electrochemical characteristics of the Li-metal anodes will be described in more detail in the following sections.

**Figure 3:**
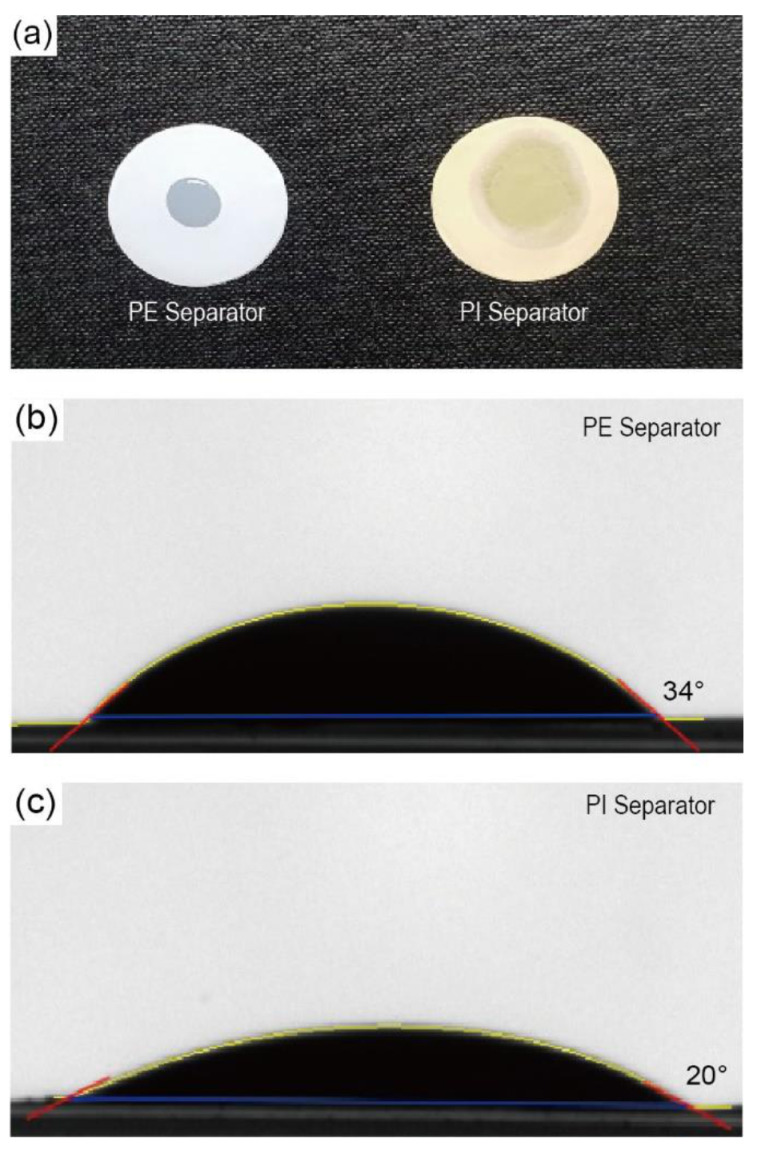
(**a**) A droplet of liquid electrolyte (a mixture of 1.15 M LiPF_6_ in ethylene carbonate/ethyl methyl carbonate (EC/EMC) (3/7 by vol.)) was dropped on the surfaces of the bare PE and porous PI separators, and the contact angle of the liquid electrolyte droplet was measured for (**b**) bare PE separator and (**c**) porous PI separator.

To investigate the wettability of separators, the droplet size and contact angle were observed after dropping droplets of the liquid electrolyte (150 μL of 1.15 M LiPF_6_ in EC/EMC (3/7, by vol.)) onto both types of separators (bare PE and porous PI separators). The droplets on the porous PI separators quickly spread over the separators. In contrast, the original size of the liquid electrolyte droplet on the bare PE separators was retained without significant changes (Figure 3a). The contact angle of the bare PE separators and porous PI separators was 34° and 20°, respectively (Figure 3b,c). These results suggest that porous PI separators have much better wettability for liquid electrolytes than bare PE separators. The improved wettability of porous PI separators is attributed to the polar moieties of PI, such as ester groups (–COO–), carboxylic groups (–COOH), and amide groups (–CONH–). In summary, the improved wetting ability of porous PI separators compared to that of bare PE separators is the main reason for the enhanced ionic conductivity and liquid electrolyte absorption of the porous PI separators.

Ionic conductivity is closely related to the total number of Li ions migrating within the separators. Owing to the improved wetting ability of the porous PI separators, they exhibited higher uptake of liquid electrolytes than the PE separators, resulting in higher ionic conductivity of the porous PI separators (Table 1). The ionic conductivity and uptake amount of porous PI separators were improved by 43% (porous PI separators = 0.931 mS cm^−1^, PE separators = 0.651 mS cm^−1^) and 200% (porous PI separators = 229.4 wt%, PE separators = 76.8 wt.%), respectively, compared to those of PE separators.

**Figure 4:**
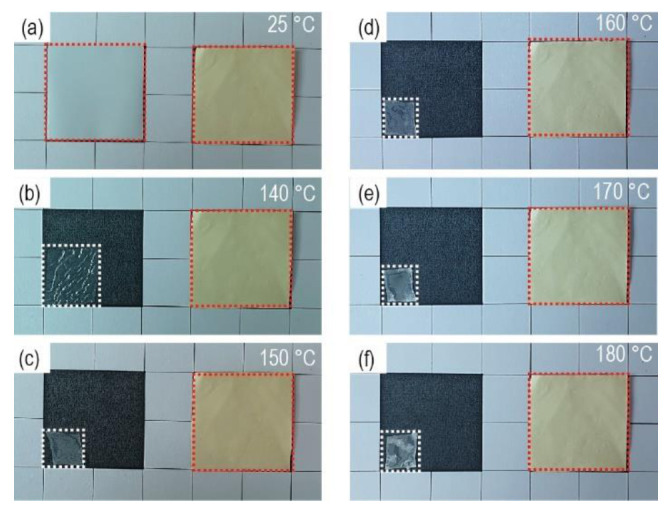
Dimensional changes of the separators (original size = 3 × 3 cm) exposed to different temperatures of (**a**) 25 °C, (**b**) 140 °C, (**c**) 150 °C, (**d**) 160 °C, (**e**) 170 °C, and (**f**) 180 °C (left = bare PE separators, right = porous PI separators).

The thermal stability of the separators was investigated by measuring the dimensional changes at high temperatures (between 140 and 180 °C) for 30 min (Figure 4). For each temperature storage, fresh separators without thermal damage were used. Both types of separators were cut into the same size (3 × 3 cm), and the dimensional changes were observed. When bare PE separators were exposed to 140 °C, they were reduced to ~50% of their original size. In the battery system, the cathodes and anodes make direct contact with the area where the separators are reduced, called “hard-short” [38]. Therefore, the separators that do not undergo dimensional changes at high-temperature exposure are vital to improving the safety of the battery system. Considering these results, it is believed that porous PI separators, which retained their original dimensions even at 180 °C exposure, are promising separator systems for high energy-density battery systems, especially those requiring high safety. Thermogravimetric analysis of PI separators supports the thermal stability of PI separators (Figure S2 in Supplementary Materials).

**Figure 5:**
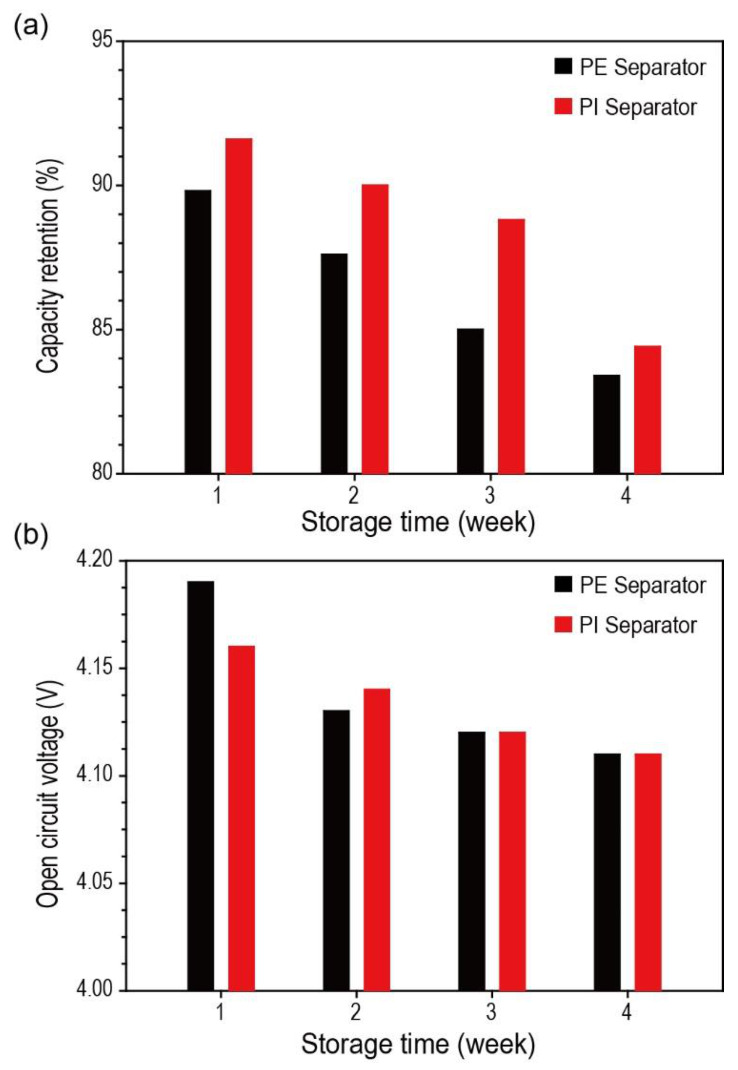
(**a**) Discharge capacity and (**b**) open-circuit voltage (OCV) of the fully charged Li half cells over storage time (1, 2, 3, and 4 weeks at 25 °C).

The effect of porous PI separators on self-discharge and open-circuit voltage (OCV) preservation was investigated. Porous PI separators demonstrated improved self-discharge preservation ability compared with bare PE separators (Figure 5a). Voltage profiles of Li metal half cells over the storage period are demonstrated in Figure S3 (in Supplementary Materials). The fully charged Li-metal half cells comprising porous PI separators showed improved discharge capacity of 2.00% in the first week, 2.74% in the second week, 4.47% in the third week, and 1.20% in the fourth week compared with those of the bare PE separators. The origin of self-discharge of the battery system was classified according to several factors [39,40]: (1) Internal electron leakage due to the partial electronic conductivity of electrolytes or other internal shorts, (2) external electron leakage from the poor isolating properties of the battery system such as battery seals or gasket, (3) electrode/electrolyte reactions, (4) partial dissolution of the electrode active materials, (5) electrode passivation by decomposition products (absorbed gases or insoluble species), (6) electrode isolation from current collectors, and (7) electrolyte leakage and internal pressure built up.

Assuming that the battery systems and storage conditions were the same, the type of separators must be an essential factor affecting the discharge capacity of the Li metal in half cells. Although polyolefin separators (i.e., polyethylene and polypropylene) are widely employed in commercialized Li secondary batteries, there was a concern regarding the oxidation of the polyolefin separators, which could deteriorate the battery system in a fully charged condition [41,42]. To demonstrate this, we investigated the chemical composition changes of both separators (bare PE and porous PI separators) after the self-discharge test. However, we did not observe any significant differences before and after the test. Consequently, it is believed that electrolyte leakage (the 7th factor listed above) is an essential factor determining the self-discharge preservation ability of Li metal half cells in our experiments, because electrolyte leakage is closely related to the wetting ability of separators. If the separators have an excellent wetting ability for liquid electrolyte, the amount of electrolyte leakage during storage will be reduced, and vice versa.

As shown in Figure 5a, compared with PE separators, the porous PI separators, exhibiting better wettability, demonstrated improved self-discharge preservation ability. The OCV results of the stored fully charged Li-metal half cells support this conclusion. The OCV of Li metal half cells comprising different types of separators (bare PE and porous PI separators) was almost the same after storage (Figure 5b). This implies that there was no electrochemical damage to the active materials, which has a significant effect on the OCV.

**Figure 6:**
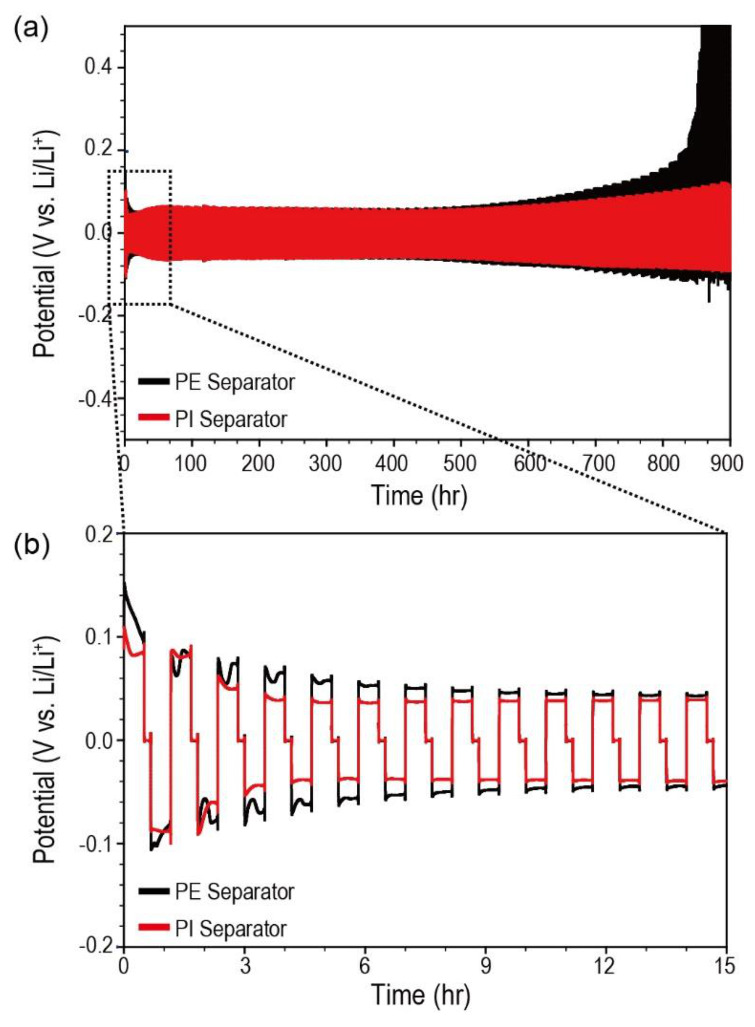
(**a**) Potential profiles of Li/Li symmetric cells containing different types of separators (bare PE separators and porous PI separators) performed based on an iterative loop [+0.5 mA cm^−2^ (30 min) → Rest (10 min) → −0.5 mA cm^−2^ (30 min) → Rest (10 min)]. (**b**) Magnified potential profiles of Li/Li symmetric cells corresponding to Figure 6a.

To investigate the effect of porous PI separators on Li-metal anodes, we assembled Li/Li symmetric cells and observed the potential profiles during the Li stripping/plating processes (Figure 6). In the beginning, both Li/Li symmetric cells containing different types of separators (Li metal/PE separator/Li metal and Li metal/porous PI separator/Li metal) demonstrated a typical dual-peaking shape. This “dual-peaking” behavior was caused by cell polarization, which (1) first decreased from the maximum value, (2) reached the minimum value, (3) increased to the local maximum, and (4) dropped again. The dual-peaking behavior is attributed to the Li-dendrite formation on the Li-metal surface [7,43]. Unlike Li/Li symmetric cells containing PE separators, the dual-peaking behavior of Li/Li symmetric cells containing porous PI separators disappeared after the first plating process, resulting in a smooth polarization profile during subsequent cycles. Furthermore, the polarization intensity of Li/Li symmetric cells containing porous PI separators was smaller than that of the PE separators. Thus, porous PI separators reduce Li-dendrite formation during the stripping/plating processes.

**Figure 7:**
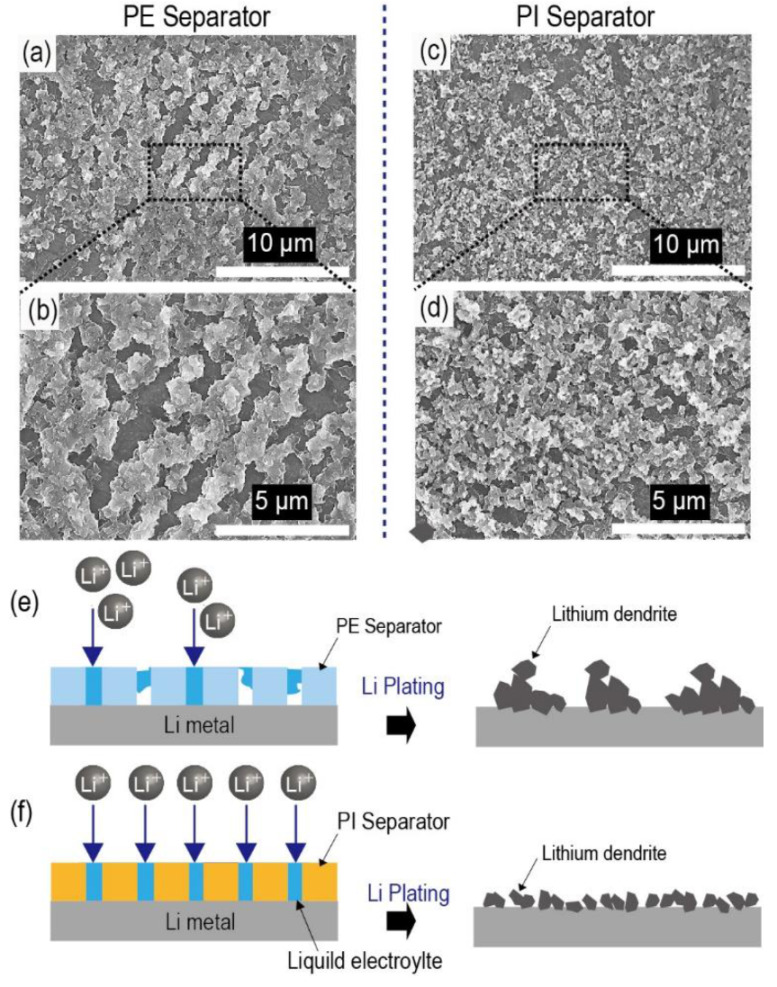
Scanning electron microscopy (SEM) images of the Li-metal surface after Li plating (0.5 mA cm^−2^ for 30 min), disassembled from the Li/Li symmetric cells containing different types of separators ((**a**,**b**) bare PE separators and (**c**,**d**) porous PI separators). Schematic figures of the Li plating mechanism according to the types of separators: (**e**) bare PE separators and (**f**) porous PI separators.

After Li plating (0.50 mA cm^−1^ for 30 min), both types of Li/Li symmetric cells (containing PE separators and porous PI separators, respectively) were disassembled, and the Li-metal surface was observed. In the case of PE separators, large-sized Li dendrites covered the Li-metal surface in certain places (Figure 7a,b). In contrast, the entire area of the Li metal of the porous PI separators was covered with much smaller and uniform Li dendrites (Figure 7c,d). Because Li dendrites originate at the point of the Li nuclei on the Li-metal surface, it can be deduced that the smaller and uniform Li dendrites of the porous PI separators are due to the uniform current distribution across the entire Li-metal surface (Figure 7e,f). Owing to the improved wetting ability of porous PI separators, more uniform Li nuclei can form on the Li-metal surface, resulting in a lower current density for each Li nucleus during Li plating under the same charging capacity conditions. This result agrees well with our suggestion that the uniform current distribution is closely related to the improved wetting ability of porous PI separators for the liquid electrolytes [4,17].

**Figure 8:**
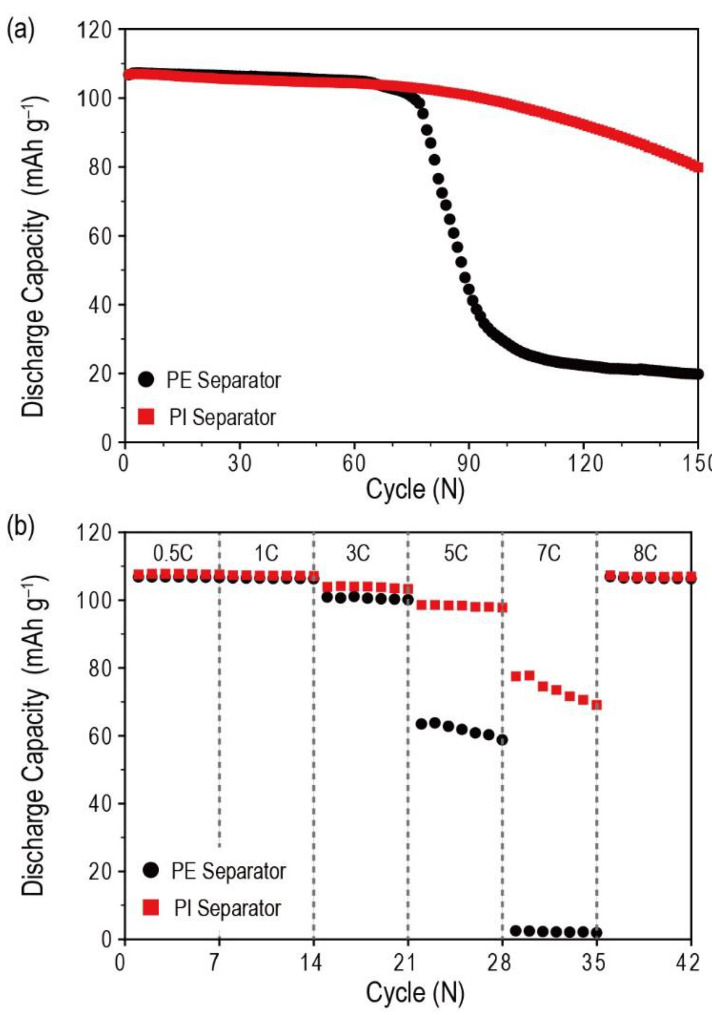
Electrochemical performance including (**a**) cycle performance (charging and discharge current density = 1C (1.2 mA cm^−2^)) and (**b**) rate capability (charging current density = C/2 (0.6 mA cm^−2^), discharging current densities varied from C/2 (0.6 mA cm^−2^) to 7C (8.4 mA cm^−2^)) of Li half cells containing different types of separators (bare PE and porous PI separators).

To investigate the effect of porous PI separators, the electrochemical performance of Li-metal anodes, cycle performance, and rate capability of Li metal half cells (LiCoO_2_/separator/Li metal) containing different types of separators (PE separators and porous PI separators) were examined. Li-metal half cells containing porous PI separators demonstrated significantly improved cycle performance compared with PE separators (Figure 8a). The former maintained 74.4% of the initial discharge capacity (initial discharge capacity = 106.8 mAh g^−1^, discharge capacity at 150th cycle = 79.4 mAh g^−1^), whereas the latter showed a sudden drop after the 75th cycle; Li-metal half cells containing PE separators maintained 18.5% of the initial discharge capacity (initial discharge capacity = 106.7 mAh g^−1^, the discharge capacity at 100th cycle = 28.7 mAh g^−1^_,_ discharge capacity at 150th cycle = 19.7 mAh g^−1^). Similar to the cycle performance results, Li-metal half-cells containing porous PI separators also showed significantly improved rate capability compared with the PE separators (Figure 8b). At the 7C-rate condition (8.32 mA cm^−2^), porous PI separators showed 72.1% of the initial discharge capacity (initial discharge capacity at C/5 rate = 107.6 mAh g^−1^, discharge capacity at 7C-rate = 77.53 mAh g^−1^). In contrast, bare PE showed only 2.26% of the initial discharge capacity (initial discharge capacity at C/5 rate = 106.8 mAh g^−1^, discharge capacity at 7C-rate = 2.4 mAh g^−1^). After returning to the C/2-rate condition at the 36th cycle, both Li-metal half cells containing PE separators and porous PI separators demonstrated similar discharge capacity as the initial discharge capacity at the 1st cycle. Thus, the reduced discharge capacity is attributed to kinetic reasons, not active material loss or decomposition. As discussed in the cycle performance results, the improved wetting ability of the separator can facilitate Li-ion migration within the porous structure of the battery system, resulting in improved rate capability [4,17,44].

If the Li dendrites touched the counter cathodes, the cell failed catastrophically, generating heat and flame called “hard-short.” Given the improved electrochemical performance of PI separators without hard-short, it can be deduced that porous PI separators have adequate physical properties (i.e., porosity and tortuosity) and can act as physical barriers [36,38,45,46].

## 4. Conclusions

We developed porous PI separators for Li-metal electrodes using the HIPE technique with a water-soluble PI precursor solution; the process is simple and environmentally friendly. In comparison with commercialized PE separators, porous PI separators have superior thermal stability and wetting ability. In addition, the performance of the porous PI separators was significantly improved over that of PE separators in terms of both physical (e.g., improved electrolyte uptake and ionic conductivity) and electrochemical properties (e.g., maintained stable self-discharge capacity and OCV properties after storage, improved cycle performance, and rate capability). Large-scale electric devices requiring high energy density, such as EVs and ESSs, must be able to meet high standards of safety, price competitiveness, and environmental conservation. Given these points, we believe that the porous PI separators that meet all these conditions are a promising candidate to replace conventional PE separators in future battery systems.

## Figures and Tables

**Table 1:** Physical properties of bare PE separators and porous PI separators.

	Thickness (μm)	Bulk Resistant (ohm)	Ionic Conductance (S)	Ionic Conductivity (mS cm^−1^)	Uptake Amount (%)
PE Separator	20	1.207	0.829	0.651	76.8
PI Separator	20	0.845	1.185	0.931	229.4

## Supplementary Materials

The following are available online at https://www.mdpi.com/2079-4991/10/10/1976/s1, Figure S1: (a) Surface and (b) cross-sectional scanning electron microscopy (SEM) images of the PI sponge before pressing. Table S1: Mercury porosimetry (AutoPore V) analysis of the PI sponge before pressing. Figure S2: (a) TGA Thermograph of PMDA-DMBZ based PI porous separator and cross-sectional SEM images of (b) pristine PI separator and c) after 250 °C heat treatment. Figure S3. Voltage profiles of stored Li metal half cells corresponding to Figure 5.
