# Supplementary material for: Highly Stable Porous Polyimide Sponge as a Separator for Lithium-Metal Secondary Batteries
Source: Nanomaterials (Basel). 2020 Oct 6;10(10):1976. doi: 10.3390/nano10101976 (PMC7600698; doi:10.3390/nano10101976)
Supplement: Supplementary file 1 [file nanomaterials-10-01976-s001.pdf]

Supplementary Materials: Highly stable porous polyimide sponge as a separator for lithium-metal secondary batteries

Junyoung Choi [1,2,3,†], Kwansoo Yang [2,3,†], Hyeon-Su Bae [1], Isheunesu Phiri [1], Hyun Jeong Ahn [2,3], Jong Chan Won [2,3], Yong Min Lee [4,*], Yun Ho Kim [2,3,*] and Myung-Hyun Ryou [1,*]

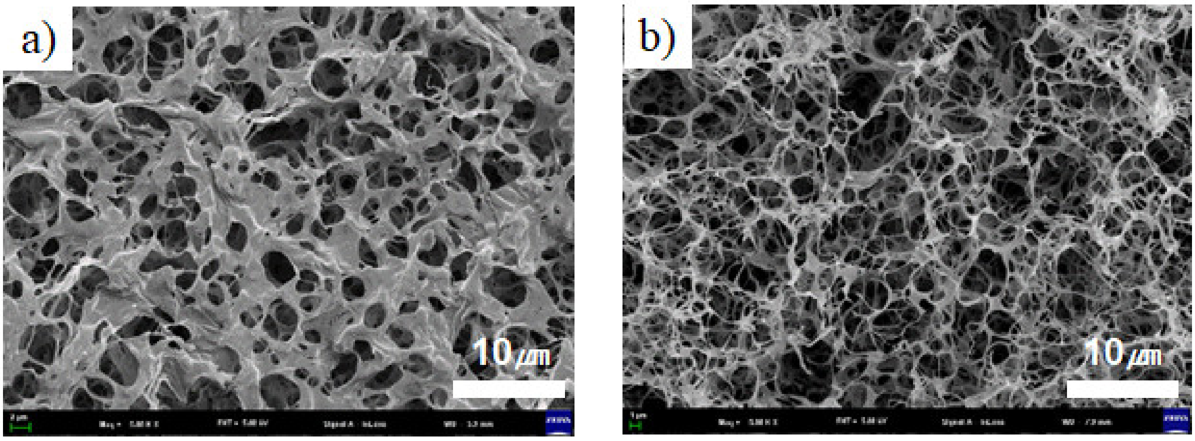

Figure S1. (**a**) Surface and (**b**) cross-sectional scanning-electron microscopy (SEM) images of PI sponge before pressing.

Table 1. Mercury porosimetry (AutoPore V) analysis of PI sponge before pressing.

Porosity (%)	Bulk density (g mL^{-1})	Apparent density (g mL^{-1})	Median pore diameter (µm)	Average pore diameter (µm)
94.9	0.0153	0.4810	0.19766	4.5998

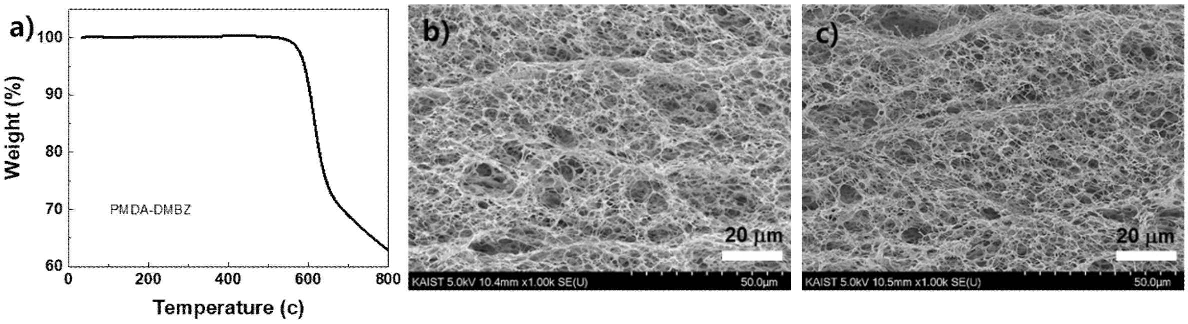

Figure S2. (**a**) TGA Thermograph of PMDA-DMBZ based PI porous separator and cross-sectional SEM images of (**b**) pristine PI separator and (**c**) after 250 °C heat treatment.

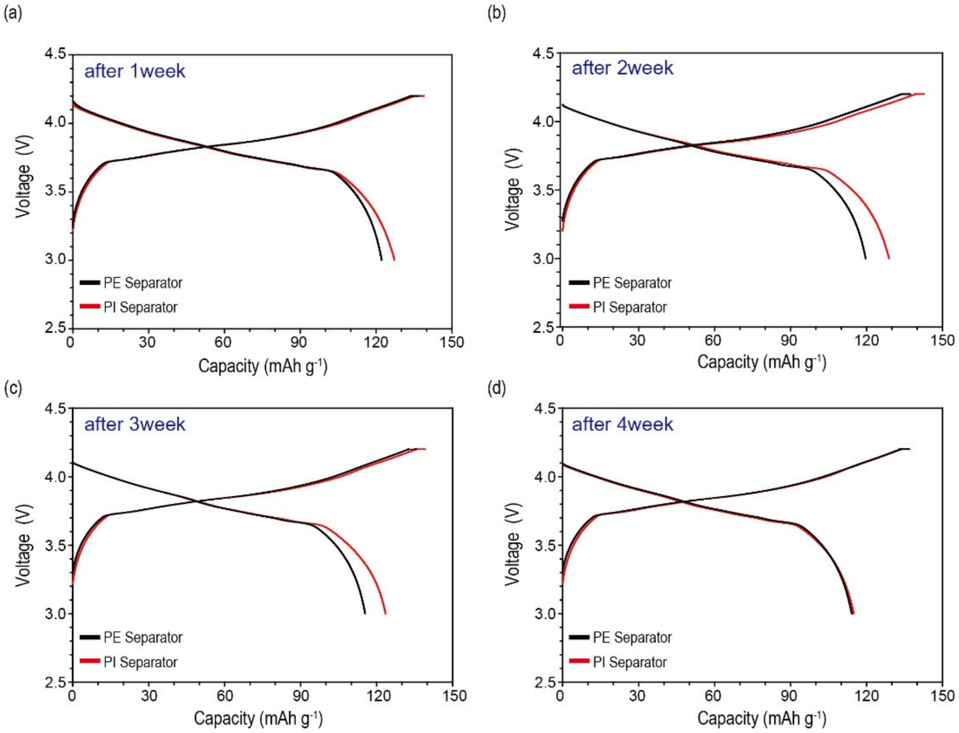

Figure S3. Voltage profiles of stored Li metal half cells corresponding to Figure 5.
